# The Association of Hypoglycemia Assessed by Continuous Glucose Monitoring With Cardiovascular Outcomes and Mortality in Patients With Type 2 Diabetes

**DOI:** 10.3389/fendo.2019.00536

**Published:** 2019-08-06

**Authors:** Wei Wei, Shi Zhao, Sha-li Fu, Lan Yi, Hong Mao, Qin Tan, Pan Xu, Guo-liang Yang

**Affiliations:** ^1^Department of Endocrinology, Tongji Medical College, The Central Hospital of Wuhan, Huazhong University of Science and Technology, Wuhan, China; ^2^Department of Cardiology, Tongji Medical College, The Central Hospital of Wuhan, Huazhong University of Science and Technology, Wuhan, China; ^3^Department of Neurology, Tongji Medical College, The Central Hospital of Wuhan, Huazhong University of Science and Technology, Wuhan, China; ^4^Department of Information, Tongji Medical College, The Central Hospital of Wuhan, Huazhong University of Science and Technology, Wuhan, China

**Keywords:** T2DM, hypoglycemia, CGM system, MACE, all-cause mortality

## Abstract

**Objective:** Hypoglycemia has been shown to promote inflammation, a common pathogenic process, in many chronic health conditions including diabetes and cardiovascular disease. The aim of this study was to investigate the association of hypoglycemia, assessed by continuous glucose monitoring (CGM) with major adverse cardiovascular event (MACE) outcomes and all-cause mortality.

**Methods:** A retrospective cohort study was conducted with 1,520 patients with type 2 diabetes mellitus (T2DM). The severity of hypoglycemia event was assessed by CGM system.

**Results:** Three hundred and forty-seven participants experienced hypoglycemia events (323 with mild hypoglycemia and 24 with severe hypoglycemia). A fraction of 72.62% hypoglycemia was asymptomatic. During a median follow-up of 31 months, 380 participants reached the primary outcome of MACE (61 cardiovascular death, 50 non-fatal myocardial infarction [MI], 116 non-fatal stroke, 153 unstable angina requiring hospitalization), 80 participants died before the end of the study. In multivariate Cox regression models, hypoglycemia was associated with cardiovascular death (HR 2.642[95CI% 1.398–4.994]), non-fatal stroke (HR 1.813 [95CI% 1.110–2.960]) and all-cause mortality (HR 1.960 [95 CI% 1.124- 3.418]) after the full adjustment. Hypoglycemia was not associated with non-fatal MI and unstable angina. The HR of severe hypoglycemia was higher than mild hypoglycemia for cardiovascular death. Patients with symptomatic and asymptomatic hypoglycemia had similar MACE outcomes and all-cause mortality.

**Conclusions:** CGM is effective to detect asymptomatic and nocturnal hypoglycemia. Hypoglycemia is associated with an increased risk of non-fatal stroke, cardiovascular related death, and total mortality. The cardiovascular mortality is dose-dependent on the severity of hypoglycemia.

## Introduction

Chronic low grade inflammation is a common feature underlying many chronic diseases and conditions such as insulin resistance and cardiovascular disease. Studies have suggested that hypoglycemia is able to promote inflammatory processes in patients with or without diabetes (1, 2). Hypoglycemia is a frequent adverse effect of anti-diabetic therapy in diabetic patients, and severe hypoglycemia has been identified as a potential risk factor for cardiovascular events in patients with type 2 diabetes mellitus (T2DM). Previous studies have reported an increased hazard ratio (HR) for adverse cardiovascular outcomes and total mortality in diabetic patients with hypoglycemia (3–6). However, these conclusions were drawn from the secondary analyses of randomized clinical trials (7, 8) or retrospective analyses of medical claims databases (9, 10), which a clear definition of hypoglycemia was lacking. Continuous glucose monitoring (CGM) system is an effective tool for the assessment of hypoglycemic status, particularly for the asymptomatic nocturnal hypoglycemia (11). Using CGM system, researchers found out that hypoglycemia is more common in T2DM patients than previously thought (12), and the frequency of hypoglycemia in those previous studies may be underestimated.

In the present study, using glucose data collected from CGMs, we investigated the relationships of hypoglycemia with total mortality and major adverse cardiovascular events (MACE), including non-fatal stroke, non-fatal myocardial infarction (MI), unstable angina leading to hospitalization, and cardiovascular death.

## Materials and Methods

### Subjects

In this retrospective cohort study, we recruited 1,520 patients admitted to our hospital between January 2013 and December 2017.

The inclusion criteria were: (1) T2DM according to 2013 American Diabetes Association standards (13). Patients with other types of diabetes, such as type 1 diabetes, gestational diabetes, and secondary diabetes were excluded from the study. (2) Patients without the acute phase of illness, such as acute coronary syndrome, uncontrolled infection, etc. (3) CGM (iPro®2 CGM System Gold, Medtronic MiniMed, Inc., Northridge, CA) was used to monitor blood glucose within 3 days of admission without changing medications before the patients were discharged from hospital.

The CGM devices used in this study continuously measured the glucose level in the interstitial fluids within the range of 2.2–22.2 mmol/L (40–400 mg/dL). The glucose level was determined every 5 min, 288 times maximum per day. The CGM system was calibrated with blood glucometer measurements (ACCU-CHEK® Aviva, Roche, Mannheim, Germany) four times daily according to the manufacturer's instruction. Participants were instructed to keep a diary about the occurrence of hypoglycemic symptoms. The CGM system used in our study measured interstitial glucose in a blinded manner, and data analysis was performed after disconnection of the device.

This clinical study was approved by the ethics committee boad of The Central Hospital of Wuhan. All participants had signed the informed consent form during the enrollment and before the start the study.

### Clinical Data Collection

A clinical data warehouse was created as a collaborative program between Wuhan Central Hospital (Wuhan, China) and Shanghai Lejiu Healthcare Technology Co., Ltd. Data were extracted every 24 h by Lex Clinical Data Application 3.2 (Shanghai Lejiu Healthcare Technology Co., Ltd) from the Hospital Health Information System (HIS) to a designated clinical data warehouse including admission/transfer/discharge, laboratory orders/results, medication orders, discharge summary, administration events, flow sheet entries, procedures, medical reports, etc. All original unstructured data (i.e., pathology report, radiology report, admit/discharge summary etc.) were exclusively converted to a uniformed structured format. Core elements of the data warehouse were completely de-identified so that all queries and analytics could be carried out without exposing the confidential health data, allowing the investigators with sufficient privilege to re-identify data. Lex Clinical Data Application 3.2 was a self-service data access tool designed to query the clinical data warehouse and return tabular data for analysis and visualization.

### Definition of Hypoglycemia

A hypoglycemia event was defined as an interstitial glucose level below 3.9 mmol/L (70 mg/dl) for at least 15 min and recovery when the interstitial glucose concentration had been continuously above the threshold for 15 min or more (11). Percent time at interstitial glucose level below 3.9 mmol/L was evaluated. A severe hypoglycemia event was defined as cognitive impairment requiring external assistance for recovery. A mild hypoglycemia event was defined as a glucose level below 3.9 mmol/L without cognitive impairment and external assistance for recovery. Nocturnal hypoglycemia was defined as an episode occurring between 00:00 a.m. and 06:00 a.m.

### Follow-Ups and Outcomes

After discharging from the hospital, participants were invited to join the out-patient blood glucose management system, which was run by the professional medical staff. Routine self-blood glucose (at least four times per week) and HbA1c (every 3 month) monitoring were demanded in order to know the glucose control condition and clinical medication. The median duration of follow-ups was 31 months (inter-quartile range, 22–56). Eighteen patients (1.18%) with new episodes of severe hypoglycemic events during follow-up were excluded from the study, and 41 (2.70%) patients were lost before follow-ups due to various reasons.

The primary outcome was the first occurrence of an adjudicated MACE, including non-fatal MI, non-fatal stroke, cardiovascular death, and unstable angina leading to hospitalization. The secondary outcome was death of any cause. The diagnoses of MACE outcomes were ascertained according to the hospitalization records, discharge summary and certification of death, which were adjudicated by an independent committee. The members of the committee were from the cardiovascular and neurology departments of our hospital, who were unaware of the CGM results. Follow-up time was calculated from the date of hypoglycemia event to the onset date of the MACE event, death, or end of study (31 August 2018). Cause of death was classified as cardiovascular death and all other causes of death.

### Statistical Analysis

The differences between groups were compared using *t-*test for continuous variables and Chi-square test for categorical data. Cox proportional models were used to evaluate the association between hypoglycemia and either MACE or all-cause mortality. We progressively adjusted the models for potential confounders. Model 1 was a crude model. Model 2 included age, sex, eGFR, HbA1c, BMI, and duration of diabetes. Model 3 included all variables in model 2 plus smoking status, alcohol history, past medical history (hepatic disease, renal disease, malignancy, coronary heart disease, and stroke), all diabetic medications (insulin, sulfonylureas, metformin, alpha-glucosidase inhibitors, pioglitazone, glinides, and DPP-4 inhibitors), hypertension medication, lipid-lowering medication, and antiplatelet agents.

For further analysis, we evaluated the risk of MACE outcomes and total mortality according to the severity of hypoglycemia and the appearance of hypoglycemic symptoms. The severity of hypoglycemia was classified into three categories: no hypoglycemia, mild hypoglycemia, and severe hypoglycemia. The group of no hypoglycemia served as the reference group, and adjusted for models described before. The survival curves of the three groups were estimate by Kaplan-Meier method, and the homogeneity between survival curves was tested by log-rank test.

All analyses were performed using State software (version 13.1, Stata Corp, College Station, TX). *P* < 0.05 was considered as statistical significance.

## Result

### Characteristics of Study Population

Baseline characteristics of the study population were presented in Table 1. The total number of patients included in the study was 1,520, with 347 (22.83%) patients experiencing hypoglycemic events. A total of 1,028 hypoglycemic events were recorded, corresponding to 250 h in hypoglycemia status. Of all the hypoglycemia, 24 patients were with severe hypoglycemia and 323 with mild hypoglycemia. The overall fraction of asymptomatic hypoglycemia was 72.62%, and the fraction of nocturnal hypoglycemia was 44.67%.

**Table 1:** Baseline characteristics of the study population (*n* = 1,520).

**Outcome**	**Number of patients**
**Severity of hypoglycemia**	
No hypoglycemia	1173 (77.17%)
Mild hypoglycemia	323 (21.25%)
Severe hypoglycemia	24 (1.58%)
**Numbers of hypoglycemia events**	
1	94 (27.09%)
2	87 (25.07%)
3	66 (19.02%)
4	40 (11.53%)
≥5	60 (17.29%)
**Symptoms of hypoglycemia**	
Asymptomatic hypoglycemia	265 (76.37%)
Symptomatic hypoglycemia	82 (23.63%)
**Time of hypoglycemia**	
Nocturnal hypoglycemia	155 (44.67%)
Diurnal hypoglycemia	192 (55.33%)
**MACE outcomes**	380
Non-fatal myocardial infraction	50 (13.16%)
Non-fatal stroke	116 (30.53%)
Cardiovascular death	61 (16.05%)
Unstable angina requiring hospitalization	153 (40.26%)
**All-cause mortality**	80

*Dates are presented as numbers (percentages)*.

*MACE, major adverse cardiovascular event*.

Compared to patients without hypoglycemia, those who experienced hypoglycemia were significantly older, had a longer duration of diabetes, and a lower eGFR. They were more likely to have experienced hypoglycemia events previously. They also took less anti-diabetic medications of metformin, pioglitazone, and DPP-4 inhibitors; meanwhile they were more often treated with insulin and anti-platelet agents (Table 2). Patients with severe hypoglycemia were even older, and more likely to be a smoker. They experienced hypoglycemia earlier than those with mild hypoglycemia (Supplementary Table 1).

**Table 2:** Clinical characteristics of participants by the occurrence of hypoglycemia.

	**No hypoglycemia** ***n* = 1,173**	**Hypoglycemia** ***n* = 347**	***P-*value**
Age, years	58.59 ± 11.26	62.27 ± 11.58	<0.001
Gender, Male (*n* %)	592 (50.5)	191 (55)	0.142
Diabetes duration, years	6.46 ± 6.00	7.78 ± 7.37	0.002
Mean glucose of CGM, mmol/L	8.97 ± 2.17	7.81 ± 2.06	<0.001
SD of CGM, mmol/L	2.64 ± 1.30	3.29 ± 1.71	<0.001
FPG, mmol/L	9.15 ± 3.81	8.36 ± 3.86	0.001
HbA1c, %	8.19 ± 2.10	7.73 ± 1.96	<0.001
BMI, kg/m^2^	24.50 ± 2.88	24.75 ± 2.90	0.173
eGFR	95.18 ± 20.29	89.09 ± 21.78	0.001
TG, mmol/L	1.83 ± 1.80	1.84 ± 1.58	0.924
TC, mmol/L	4.58 ± 1.06	4.62 ± 1.02	0.616
HDL, mmol/L	2.61 ± 0.85	2.60 ± 0.84	0.750
LDL, mmol/L	1.13 ± 0.33	1.14 ± 0.29	0.844
Systolic blood pressure, mmHg	130.46 ± 17.03	132.00 ± 19.84	0.154
Diastolic blood pressure, mmHg	78.67 ± 9.78	77.70 ± 11.09	0.116
Smoking status	340 (29)	102 (29.4)	0.893
Alcohol history	151 (12.9)	59 (17)	0.052
Previous hypoglycemia	73 (6.2)	57 (16.4)	<0.001
History of hepatic disease	160 (13.6)	48 (13.8)	0.624
History of renal disease	32 (2.7)	23 (6.6)	0.002
History of malignancy	29 (2.5)	12 (3.5)	0.089
History of coronary heart disease	191 (16.3)	66 (19.0)	0.254
History of stroke	79 (6.7)	26 (7.5)	0.630
**Diabetic Complication**			
Diabetic nephropathy	331 (28.2)	106 (30.5)	0.418
Diabetic retinopathy	283 (24.1)	91 (26.2)	0.436
Diabetic peripheral neuropathy	246 (21.0)	78 (22.5)	0.551
Peripheral arterial disease	8 (0.7)	4 (1.2)	0.487
**Diabetic Medications**			
Insulin	444 (37.9)	179 (51.6)	0.000
Sulfonylureas	167 (14.2)	59 (17.0)	0.229
Metformin	407 (34.7)	55 (15.9)	<0.001
Alpha-glucosidase inhibitors	437 (37.3)	124 (35.7)	0.613
Pioglitazone	112 (9.5)	19 (5.5)	0.016
Glinides	73 (6.2)	23 (6.6)	0.802
DPP-4 inhibitors	87 (7.4)	12 (3.5)	0.009
Hypertension medication	527 (44.9)	176 (50.7)	0.058
Lipid-lowering medication	269 (22.9)	78 (22.5)	0.884
Antiplatelet agents	358 (30.5)	132 (38.0)	0.009

*Continuous variables are shown as mean ± SD. Categorical data are presented as numbers (percentages)*.

*CGM, continuous glucose monitoring; SD, standard deviation; FPG, fasting plasma glucose; BMI, body mass index; GFR, glomerular filtration rate; TG, triglyceride; TC, total cholesterol; LDL, low density lipoprotein; HDL, high density lipoprotein; DPP-4, dipeptidylpeptidse 4*.

Patients with hypoglycemia were further divided into two groups according to the appearance of hypoglycemic symptoms. Compared to patients with symptomatic hypoglycemia, patients experiencing asymptomatic hypoglycemia had lower mean glucose of CGM and smaller glycemic variability, were more likely to experience nocturnal hypoglycemia, and had longer periods of hypoglycemic events (Supplementary Table 2). The other baseline clinical characteristics of patients with symptomatic hypoglycemia were similar to those with asymptomatic hypoglycemia, except that patients experiencing asymptomatic hypoglycemia had a higher proportion of diabetic peripheral neuropathy (Supplementary Table 3).

### Association Between Hypoglycemia and MACE Outcomes

During the follow-up, 380 diabetic patients had developed MACE (61 cardiovascular deaths, 153 unstable angina requiring hospitalization, 50 non-fatal MI, 116 non-fatal strokes). Eighty patients died before the end of our study. Of the 347 patients with hypoglycemia, the median time between hypoglycemia events and MACE outcomes was 21 (inter-quartile range, 11–38) months.

The crude incidence of MACE outcomes in people with hypoglycemia was higher than the incidence in people without hypoglycemia. These estimated results still remained significant (model 2: HR 1.592, 95%CI, 1.233-2.056; model 3: HR 1.615, 95% CI, 1.239-2.106) after further adjustments for potential confounding factors.

Furthermore, we examined the findings by subtypes of MACE outcomes. Compared to patients without hypoglycemia, those with hypoglycemia had a higher rate for non-fatal stroke, cardiovascular death and total mortality (Table 3). The associations were still persistent after additional adjustment in model 2 and model 3. In the minimally adjusted models, hypoglycemia was associated with an increased risk of non-fatal MI, which was no longer observed after further adjustment (model 3: HR 1.549, 95%CI 0.768–3.124). No association with hypoglycemia was found for unstable angina requiring hospitalization in any model.

**Table 3:** Association between hypoglycemia and MACE outcomes and all-cause mortality.

	**Events/ N**			**Model 1**	**Model 2**	**Model 3**
	**With hypoglycemia**	**Without hypoglycemia**	***p***	**HR (95%CI)**	**HR (95%CI)**	**HR (95%CI)**
MACE	117/333	263/1,128	<0.001	1.501 (1.207, 1.866)	1.592 (1.233, 2.056)	1.615 (1.239, 2.106)
Cardiovascular death	23/333	38/1,128	0.006	2.033 (1.211, 3.413)	2.652 (1.433, 4.914)	2.642 (1.398, 4.994)
Unstable angina requiring hospitalization	41/333	112/1,128	0.300	1.226 (0.857, 1.753)	1.172 (0.774, 1.774)	1.218 (0.794, 1.869)
Non-fatal MI	18/333	32/1,128	0.030	1.901 (1.067, 3.389)	1.634 (0.828, 3.226)	1.549 (0.768, 3.124)
Non-fatal stroke	35/333	81/1,128	0.060	1.691 (1.144, 2.499)	1.755 (1.099, 2.803)	1.813 (1.110, 2.960)
All-cause mortality	34/333	46/1,128	<0.001	2.501 (1.605, 3.898)	2.259 (1.323, 3.858)	1.960 (1.124, 3.418)

*Hypoglycemia was modeled as a time-dependent exposure*.

*Model 1 was a crude model*.

*Model 2 included age, sex, eGFR, HbA1c, BMI, and duration of diabetes*.

*Model 3 included all variables in model 2 plus smoking status, alcohol history, past medical history (hepatic disease, renal disease, malignancy, coronary heart disease, and stroke), all diabetic medications (insulin, sulfonylureas, metformin, alpha-glucosidase inhibitors, pioglitazone, glinides, and DPP-4 inhibitors), hypertension medication, lipid-lowering medication, and antiplatelet agents*.

*MACE, major adverse cardiovascular event; MI, myocardial infarction*.

**Figure 1:**
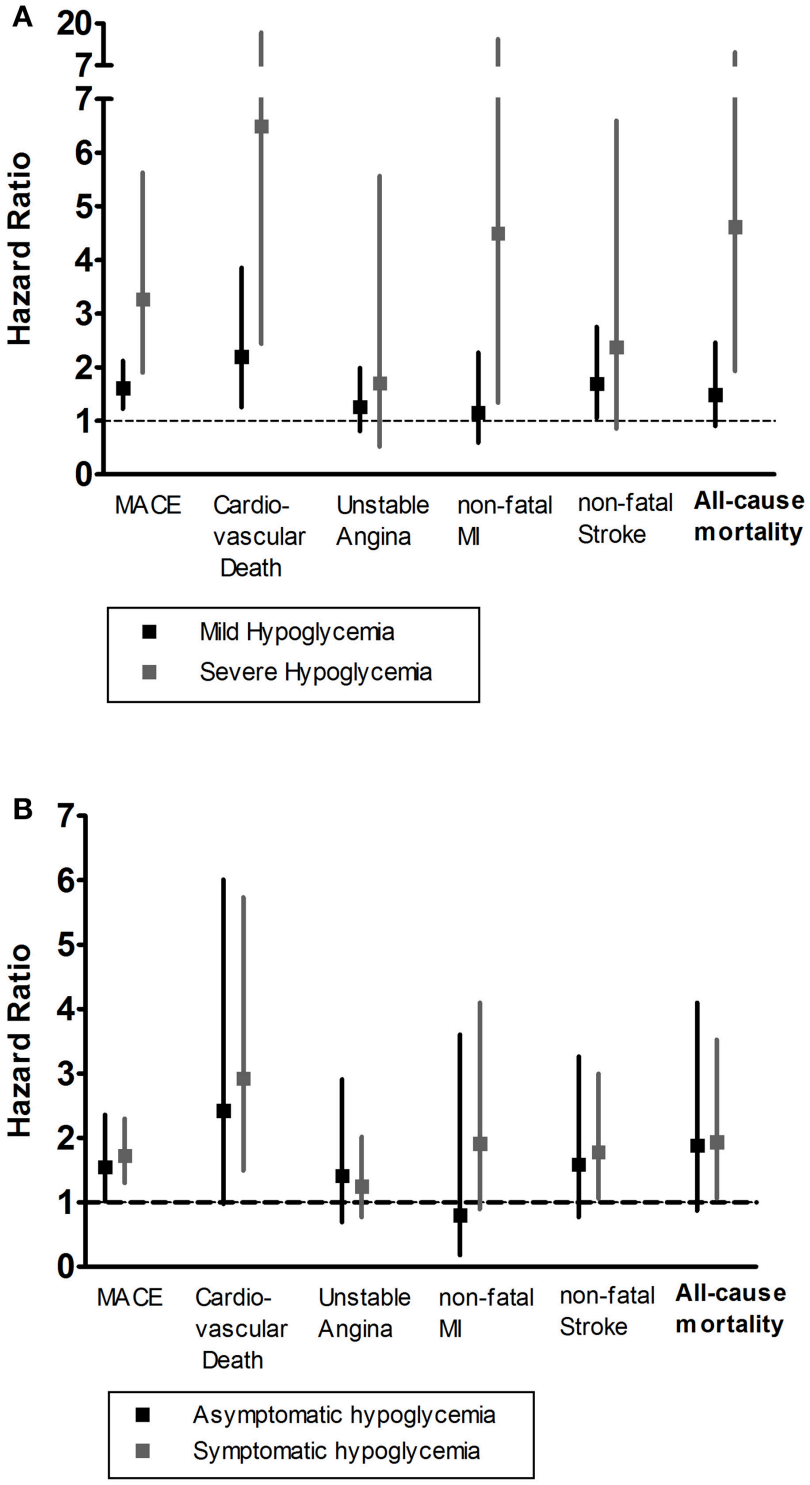
**(A)** The HR and 95% CIs of MACE outcomes and all-cause mortality according to the severity of hypoglycemia. **(B)** The HR and 95% CIs of MACE outcomes and all-cause mortality according to the appearance of hypoglycemic symptoms. The severity of hypoglycemia was classified into three categories: no hypoglycemia, mild hypoglycemia, and severe hypoglycemia. The appearance of hypoglycemic symptoms was classified into three categories: no hypoglycemia, asymptomatic hypoglycemia, and symptomatic hypoglycemia. The group of no hypoglycemia served as the reference group, and adjusted for models including age, sex, duration of diabetes, HbA1c, BMI, eGFR, past medical history, diabetes medications, hypertension medication, lipid-lowering medication, and antiplatelet agents. HR, Hazard Ratios; MACE, major adverse cardiovascular event; MI, myocardial infarction.

**Figure 2:**
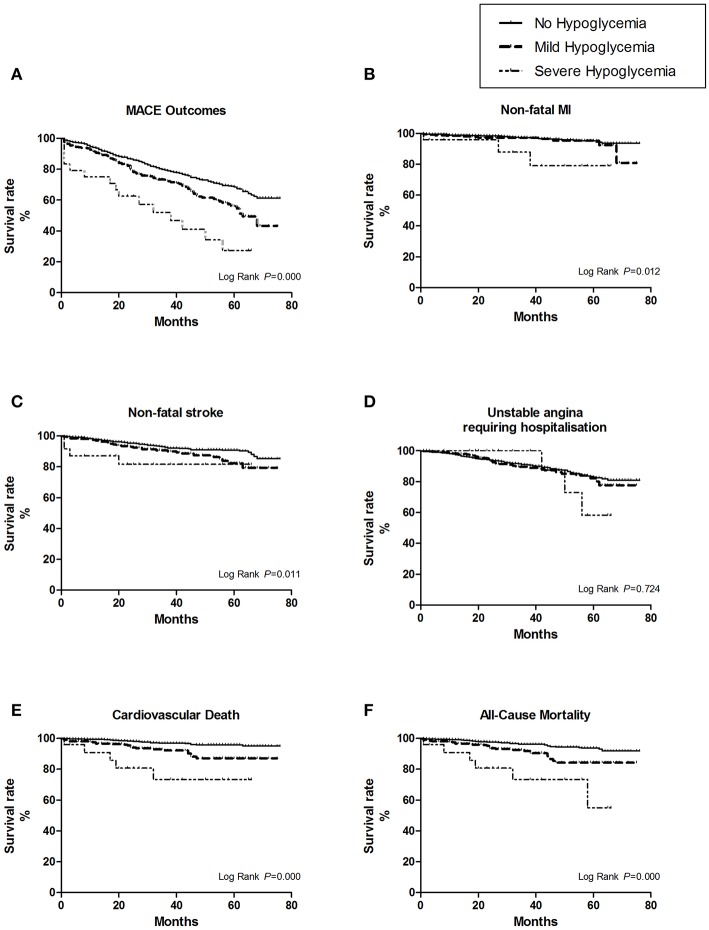
The survival curves of the hypoglycemic severity were estimate by Kaplan-Meier method, and the homogeneity between survival curves was tested by log-rank test. **(A)** For overall MACE outcomes, the risk of MACE was the highest in the first year after severe hypoglycemia (25%, 6/24). **(B)** For the subtype of non-fatal myocardial infarction (MI). **(C)** For the subtype of non-fatal stroke. **(D)** For the subtype of unstable angina leading to hospitalization. **(E)** For the subtype of cardiovascular death. **(F)** For all cause-mortality.

**Figure 3:**
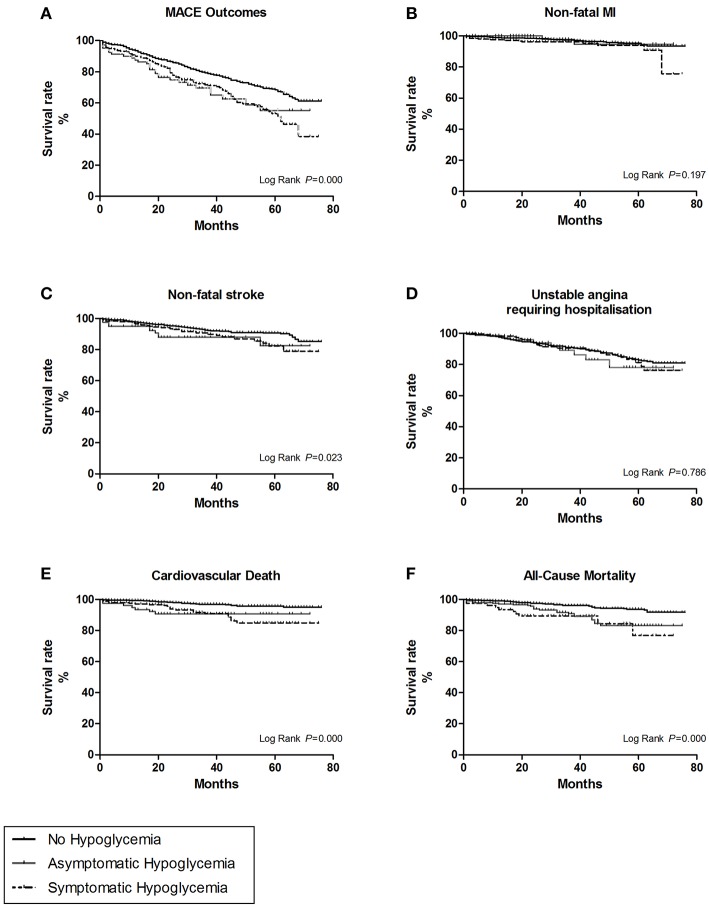
The survival curves of the hypoglycemic symptoms were estimate by Kaplan-Meier method, and the homogeneity between survival curves was tested by log-rank test. **(A)** For overall MACE outcomes. **(B)** For the subtype of non-fatal myocardial infarction (MI). **(C)** For the subtype of non-fatal stroke. **(D)** For the subtype of unstable angina leading to hospitalization. **(E)** For the subtype of cardiovascular death. **(F)** For all cause-mortality.

Patients with severe hypoglycemia had a higher risk of cardiovascular death than those with mild hypoglycemia (Figure 1A). For subtypes of MACE outcomes, the values of HRs had a trend of rising in the severe hypoglycemia group compared with those in the hypoglycemia group, but the difference did not reach statistical significance (Figure 2). Patients with symptomatic and asymptomatic hypoglycemia had similar MACE outcomes and all-cause mortality (Figures 1B, 3).

## Discussion

The results of our study showed that hypoglycemia events detected by CGM were strongly associated with subsequent MACE outcomes and all-cause mortality. This association persisted after adjustment for a wide range of confounders. Furthermore, the risk of cardiovascular death and all-cause mortality was the highest after severe hypoglycemia events during CGM monitoring, especially in the first year, suggesting that health care providers should pay particular attention to the potential for morbidity and mortality after a severe hypoglycemic event. CGMs also detected a high proportion of asymptomatic hypoglycemic events, which appeared to have a similarly effect on MACE outcomes and all-cause mortality like the symptomatic hypoglycemia.

The Action to Control Cardiovascular Risk in Diabetes (ACCORD) study reported that intensive glycemic control was associated with increased risk of cardiovascular-related death (3, 4). Since the premature closure of the ACCORD study, the hypoglycemia-related cardiovascular adverse outcomes have led to considerable debate. The impacts of hypoglycemia on cardiovascular events in diabetic patients have been evaluated in several large prospective clinical trials, which have different conclusions (5, 6, 14). Also, there were many observational studies, with inconsistent results of the association between hypoglycemia and MACE outcomes (7, 15). A subsequent meta-analysis including 10 studies suggested that severe hypoglycemia was associated with an almost two fold increased risk of cardiovascular events (17). Consistent with parts of those previous studies, the results of our analyses showed that hypoglycemia was associated with cardiovascular-related death and all-cause mortality. However, due to the insufficient cases of non-cardiovascular mortality (*n* = 19), we were unable to analyze the association between the cause-specific mortality and hypoglycemia like the Trail Comparing Cardiovascular Safety of Insulin Degludec vs. Insulin Glargine in Patients with Type 2 Diabetes at High Risk of Cardiovascular Events (DEVOTE)study (16). In analysis of other subtypes of MACE outcomes, we can only see the association between hypoglycemia and an increased risk of developing non-fatal stroke. There was only a trend between hypoglycemia and non-fatal MI and unstable angina requiring hospitalization, which contrasted with the previous analyses (10, 14).

This can be explained by the different definitions of hypoglycemia, resulting in variable frequencies of hypoglycemia across studies. For most large epidemiological studies, hypoglycemia cases were collected by self-report (5, 6) or ICD-codes from medical electronic data (9, 10, 14), which may underestimate the prevalence of hypoglycemia. In our study, a fraction of 76.37% hypoglycemic episodes were asymptomatic, which was also supported by earlier studies using CGM system (18, 19). Such a high proportion of asymptomatic hypoglycemia in diabetic patients with reduced awareness is worth our serious attention in clinical management. CGM is an effective way to detect hypoglycemia events, especially nocturnal and asymptomatic hypoglycemia, which could play an important role in reducing hypoglycemia events and is worth promoting in clinical applications.

We found some evidence of a dose-dependent relationship between the severity of hypoglycemia and cardiovascular death and all-cause mortality. Our assumption is that the cardiovascular outcomes of severe hypoglycemia may be worse than that of mild hypoglycemia. A sub-analysis of the ACCORD study found that the protective effect of recurrent mild hypoglycemia was more pronounced than severe hypoglycemia (20). It is suggested that exposure to mild hypoglycemia may offer better preparation against the adverse cardiovascular outcomes caused by severe hypoglycemia through prior blunting of sympathetic responses (21). In contrast to the findings of our study, several observational studies (22, 23) found out that mild hypoglycemia events have no association with mortality. Differences in methods of defining mild hypoglycemia may contribute to discrepancies. However, given the small number of participants with severe hypoglycemia (*n* = 24) in our study, these results may be limited by low statistical power.

The clinical management of T2DM emphasizes the importance of glycemic control to reduce the risk of chronic complications associated with diabetes (24). However, a too-intensive glucose management therapy also puts patients at increased risk of hypoglycemia, which could be life-threatening. Given the concern that hypoglycemia might be a risk factor for cardiovascular disease, avoiding hypoglycemia remains a significant goal in optimizing glucose control. Individualizing glycemic targets should be considered for people with T2DM who are at high risk for hypoglycemia.

Currently, the standard of care in clinical practice is self-monitoring of capillary blood glucose (SMBG), which only provides a single point of time measurement and often fails to detect nocturnal and asymptomatic hypoglycemia. With the ability to measure glucose levels continuously and reflect glycemic variability, CGM technology is gaining increasing interest in clinical management. Numerous studies using CGM have demonstrated significant improvements in reducing hypoglycemia (25, 26). Future incorporation of CGMs in large clinical trials may provide precise information on the severity of hypoglycemia, as well as the glucose level at the occurrence of a hypoglycemic event.

Our study has two important strengths. First, to our knowledge, this is the first study of applying CGM to reveal the relationship between hypoglycemia and the increased risk of CVD. Previous epidemiologic investigations without an accurate definition of hypoglycemia may underestimate the prevalence of hypoglycemia events. Both the severity and time of hypoglycemia can be collected precisely through the CGM system to make a precise diagnosis of hypoglycemia. Second, we were able to adjust for numerous standardized and high-quality covariates, including the duration of diabetes, personal habits (smoking and drinking), BMI, and kidney function. In most large clinical trials, the ICD-code extracted from electronic medical records may be inaccurate, possibly leading to misclassification of exposure and confounding factors.

There were several limitations in our research. Firstly, the number of events for some outcomes may limit the precision of our estimations. Secondly, our study had a relatively short duration of follow-up with a median time of 31 months, which may limit the power to detect a significant association. Thirdly, the study only recruited participants of T2DM during hospitalization, which may not be generalizable beyond this population. Finally, the retrospective nature of our study precludes the possibility to explain the direct causal effect between hypoglycemia and MACE outcomes. Thus, well-designed prospective cohort studies with the primary intention are needed to evaluate the association between hypoglycemia and cardiovascular outcomes.

In conclusion, through the analysis of glucose data collected by using CGMs, our results add to the accumulating evidence that hypoglycemia is associated with an increased risk of non-fatal stroke, cardiovascular death, and all-cause mortality. We also revealed a dose-dependent relationship between the severity of hypoglycemia and cardiovascular outcomes. Therefore, effective measurements should be taken to prevent severe hypoglycemia in patients with T2DM, especially those at high risk of cardiovascular problems.

## Data Availability

The raw data supporting the conclusions of this manuscript will be made available by the authors, without undue reservation, to any qualified researcher.

## Ethics Statement

This clinical study was approved by the ethics committee broad of The Central Hospital of Wuhan. All participants had signed the informed consent form during the enrollment and before the start the study.

## Author Contributions

WW and SZ conceived and designed the study. WW conducted the statistical analyses and wrote the manuscript. SZ provided guidance for the statistical analysis and made critical revisions to the manuscript for important intellectual content. HM provided guidance for the statistical analysis. GY collaborated with Shanghai Lejiu Healthcare Technology Co., Ltd. and took responsibility for the integrity of the data. QT and PX adjudicated the MACE outcomes and the cause of death. LY and SF took charge of the patients' follow-up.

### Conflict of Interest Statement

The authors declare that the research was conducted in the absence of any commercial or financial relationships that could be construed as a potential conflict of interest.
