# Supplementary material for: The Association of Hypoglycemia Assessed by Continuous Glucose Monitoring With Cardiovascular Outcomes and Mortality in Patients With Type 2 Diabetes
Source: Front Endocrinol (Lausanne). 2019 Aug 6;10:536. doi: 10.3389/fendo.2019.00536 (PMC6691179; doi:10.3389/fendo.2019.00536)
Supplement: Supplementary file 1 [file Data_Sheet_1.docx]

**Supplementary Table 1 -Clinical Characteristics of participants by the severity of hypoglycemia**

|  | Mild hypoglycemia  n=323 | Severe hypoglycemia  n=24 | *P* |
| --- | --- | --- | --- |
| Age, years | 61.83 ± 11.52 | 68.12 ± 10.98 | **0.010** |
| Males, % | 178 (55.1) | 13 (54.2) | 1.000 |
| Diabetes duration, years | 7.70 ± 7.41 | 8.82 ± 6.88 | 0.475 |
| Mean glucose of CGM, mmol/L | 7.69 ± 2.01 | 9.38 ± 2.17 | **< 0.001** |
| SD of CGM, mmol/L | 3.23 ± 1.67 | 4.07 ± 2.00 | **0.020** |
| FPG, mmol/L | 8.48 ± 3.95 | 6.76 ± 1.83 | **< 0.001** |
| HbA1c, % | 7.78 ± 1.98 | 6.97 ± 1.51 | **0.050** |
| BMI,kg/㎡ | 24.77 ± 2.88 | 24.35 ± 3.23 | 0.494 |
| eGFR | 89.66 ± 21.40 | 81.38 ± 25.59 | 0.072 |
| TG | 1.85 ± 1.61 | 1.70 ± 1.22 | 0.647 |
| TC | 4.63 ± 1.03 | 4.39 ± 0.89 | 0.206 |
| HDL | 2.63 ± 0.84 | 2.28 ± 0.82 | 0.819 |
| LDL | 1.14 ± 0.30 | 1.12 ± 0.19 | 0.052 |
| Systolic blood pressure, mmHg | 131.50 ± 19.32 | 138.75 ± 25.42 | 0.184 |
| Diastolic blood pressure, mmHg | 77.86 ± 10.84 | 75.54 ± 14.15 | 0.439 |
| Smoking status | 100 (31) | 2 (8.3) | **0.019** |
| Alcohol history | 55 (17.0) | 4 (16.7) | 1.000 |
| Previous hypoglycemia | 49 (15.2) | 8 (33.3) | **0.039** |
| History of hepatic disease | 43 (13.3) | 5 (20.8) | 0.352 |
| History of renal disease | 20 (6.2) | 3 (12.5) | 0.206 |
| History of malignancy | 10 (3.1) | 2 (8.3) | 0.198 |
| History of coronary heart disease | 60 (18.6) | 6 (25.0) | 0.424 |
| History of stroke | 23 (7.1) | 3 (12.5) | 0.408 |
| Diabetic complication,% |  |  |  |
| diabetic nephropathy | 99 (30.7) | 7 (29.2) | 1.000 |
| diabetic retinopathy | 86 (26.6) | 5 (20.8) | 0.637 |
| diabetic peripheral neuropathy | 70 (21.7) | 8 (33.3) | 0.206 |
| peripheral arterial disease | 4 (1.2) | 0 (0) | 1.000 |
| Diabetic Medications, % |  |  |  |
| Insulin | 168 (52.0) | 11 (45.8) | 0.673 |
| Sulfonylureas | 54 (16.7) | 5 (20.8) | 0.577 |
| Metformin | 54 (16.7) | 1 (4.2) | 0.146 |
| Alpha-glucosidase inhibitors | 113 (35.0) | 11 (45.8) | 0.377 |
| Pioglitazone | 17 (5.3) | 2 (8.3) | 0.631 |
| Glinides | 20 (6.2) | 3 (12.5) | 0.206 |
| DPP-4 inhibitors | 12 (3.7) | 0 (0) | 1.000 |
| Hypertension medication, % | 161 (49.8) | 15 (62.5) | 0.291 |
| Lipid-lowering medication, % | 72 (22.3) | 6 (25.0) | 0.800 |
| Antiplatelet agents, % | 118 (36.5) | 14 (58.3) | **0.048** |

Continuous variables are shown as mean ± SD. Categorical data are presented as numbers (percentages).

CGM, continuous glucose monitoring; SD, standard deviation; FPG: fasting plasma glucose; BMI: Body Mass Index; GFR: Glomerular Filtration Rate; TG: triglyceride; TC: total cholesterol; LDL: low density lipoprotein; HDL: high density lipoprotein; DPP-4: dipeptidylpeptidse 4.

**Supplementary Table 2 - CGM characteristics and MACE outcomes between participants with and without symptoms of hypoglycemia**

|  | **Symptomatic hypoglycemia** | **Asymptomatic hypoglycemia** |  |
| --- | --- | --- | --- |
|  | n=82 | n=265 | *P* |
| Mean glucose of CGM, mmol/L | 8.80 ± 2.18 | 7.50 ± 1.93 | **< 0.001** |
| SD of CGM, mmol/L | 3.62 ± 1.68 | 3.19 ± 1.70 | **0.047** |
| Time of hypoglycemia events, minutes | 327.09 ± 286.91 | 500.97 ± 408.536 | **< 0.001** |
| Numbers of hypoglycemia events |  |  |  |
| 1 | 19 (23.2) | 75 (28.3) | 0.593 |
| 2 | 23 (28.0) | 64 (24.2) |  |
| 3 | 14 (17.1) | 52 (19.6) |  |
| 4 | 9 (11.0) | 31 (11.7) |  |
| ≥5 | 17 (20.7) | 43 (16.23) |  |
| Time of hypoglycemia |  |  |  |
| Nocturnal hypoglycemia | 45 (54.9) | 160 (60.4) | **0.018** |
| Diurnal hypoglycemia | 37 (45.1) | 105 (39.6) |  |
| MACE outcomes | 28 (34.6) | 89 (35.3) | 1.000 |
| Non-fatal MI | 2 (2.5) | 13 (5.2) | 0.537 |
| Non-fatal stroke | 10 (12.3) | 27 (10.7) | 0.687 |
| Unstable angina requiring hospitalization | 9 (11.1) | 27 (10.7) | 1.000 |
| Cardiovascular death | 7 (8.6) | 22 (8.7) | 1.000 |
| All-cause mortality | 10 (12.3) | 24 (9.5) | 0.527 |

Continuous variables are shown as mean±SD. Categorical data are presented as numbers (percentages).

MACE: major adverse cardiovascular event; MI: myocardial infarction.

**Supplementary Table 3-Clinical Characteristics of participants by the symptoms of hypoglycemia**

|  | Symptomatic hypoglycemia  n=82 | Asymptomatic hypoglycemia  n=265 | *P* |
| --- | --- | --- | --- |
| Age, years | 63.72 ± 13.93 | 61.82 ± 10.75 | 0.195 |
| Males, % | 47 (57.3) | 144 (54.3) | 0.704 |
| Diabetes duration, years | 8.76 ± 7.24 | 7.48 ± 7.40 | 0.171 |
| Mean glucose of CGM, mmol/L | 8.80 ± 2.18 | 7.50 ± 1.93 | **< 0.001** |
| SD of CGM, mmol/L | 3.62 ± 1.68 | 3.19 ± 1.70 | **0.047** |
| FPG, mmol/L | 8.79 ± 4.18 | 8.23 ± 3.76 | 0.253 |
| HbA1c, % | 7.92 ± 1.95 | 7.67 ± 1.97 | 0.309 |
| BMI,kg/㎡ | 24.67 ± 2.88 | 24.77 ± 2.91 | 0.783 |
| eGFR | 85.63 ± 25.37 | 90.16 ± 20.47 | 0.100 |
| TG | 1.81 ± 1.34 | 1.85 ± 1.66 | 0.844 |
| TC | 4.67 ± 1.01 | 4.60 ± 1.03 | 0.568 |
| HDL | 2.62 ± 0.89 | 2.60 ± 0.83 | 0.838 |
| LDL | 1.15 ± 0.31 | 1.13 ± 0.28 | 0.638 |
| Systolic blood pressure, mmHg | 133.45 ± 19.37 | 131.55 ± 20.00 | 0.450 |
| Diastolic blood pressure, mmHg | 76.66 ± 10.99 | 78.03 ± 11.12 | 0.330 |
| Smoking status | 22 (26.8) | 80 (30.2) | 0.583 |
| Alcohol history | 14 (17.1) | 45 (17.0) | 1.000 |
| Previous hypoglycemia | 11 (13.4) | 46 (17.4) | 0.496 |
| History of hepatic disease | 13 (15.9) | 35 (13.2) | 0.584 |
| History of renal disease | 6 (7.3) | 17 (6.4) | 0.800 |
| History of malignancy | 4 (4.9) | 8 (3.0) | 0.488 |
| History of coronary heart disease | 16 (19.5) | 50 (18.9) | 0.873 |
| History of stroke | 7 (8.5) | 19 (7.2) | 0.638 |
| Diabetic complication,% |  |  |  |
| diabetic nephropathy | 26 (31.7) | 80 (30.2) | 0.786 |
| diabetic retinopathy | 19 (23.2) | 72 (27.2) | 0.566 |
| diabetic peripheral neuropathy | 27 (32.9) | 15 (19.2) | **0.015** |
| peripheral arterial disease | 1 (1.2) | 3 (1.1) | 1.000 |
| Diabetic Medications, % |  |  |  |
| Insulin | 49 (59.8) | 130 (49.1) | 0.101 |
| Sulfonylureas | 12 (14.6) | 47 (17.7) | 0.615 |
| Metformin | 10 (12.2) | 45 (17.0) | 0.387 |
| Alpha-glucosidase inhibitors | 28 (34.1) | 96 (36.2) | 0.793 |
| Pioglitazone | 4 (4.9) | 15 (5.7) | 1.000 |
| Glinides | 7 (8.5) | 16 (6.0) | 0.448 |
| DPP-4 inhibitors | 4 (4.9) | 8 (3.0) | 0.488 |
| Hypertension medication, % | 44 (53.7) | 132 (49.8) | 0.613 |
| Lipid-lowering medication, % | 17 (20.7) | 61 (23.0) | 0.763 |
| Antiplatelet agents, % | 35 (42.7) | 97 (36.6) | 0.363 |

Continuous variables are shown as mean±SD. Categorical data are presented as numbers (percentages).

CGM, continuous glucose monitoring; SD, standard deviation; FPG: fasting plasma glucose; BMI: Body Mass Index; GFR: Glomerular Filtration Rate; TG: triglyceride; TC: total cholesterol; LDL: low density lipoprotein; HDL: high density lipoprotein; DPP-4: dipeptidylpeptidse 4.
